# Zebrafish models for functional and toxicological screening of nanoscale drug delivery systems: promoting preclinical applications

**DOI:** 10.1042/BSR20170199

**Published:** 2017-06-07

**Authors:** Keon Yong Lee, Gun Hyuk Jang, Cho Hyun Byun, Minhong Jeun, Peter C. Searson, Kwan Hyi Lee

**Affiliations:** 1 Center for Biomaterials, Biomedical Research Institute, Korea Institute of Science and Technology (KIST), Seoul 02792, Republic of Korea; 2 Department of Biomedical Engineering, Korea University of Science and Technology (UST), Daejeon 02792, Republic of Korea; 3 Division of Life Sciences, School of Life Science and Biotechnology, Korea University, Seoul 02792, Republic of Korea; 4 Institute for Nanobiotechnology (INBT), Johns Hopkins University, Baltimore, MD 21218, U.S.A.; 5 Department of Materials Science and Engineering, Johns Hopkins University, Baltimore, MD 21218, U.S.A.

**Keywords:** Biodistribution, Biotoxicity, Drug delivery system, Functional evaluation, Zebrafish model

## Abstract

Preclinical screening with animal models is an important initial step in clinical translation of new drug delivery systems. However, establishing efficacy, biodistribution, and biotoxicity of complex, multicomponent systems in small animal models can be expensive and time-consuming. Zebrafish models represent an alternative for preclinical studies for nanoscale drug delivery systems. These models allow easy optical imaging, large sample size, and organ-specific studies, and hence an increasing number of preclinical studies are employing zebrafish models. In this review, we introduce various models and discuss recent studies of nanoscale drug delivery systems in zebrafish models. Also in the end, we proposed a guideline for the preclinical trials to accelerate the progress in this field.

## Introduction

Relative to the dynamic research atmosphere in the development of nanoscale drug delivery systems, the number of consumer drugs that incorporate nanotechnology is very low. Countless nanoscale drug delivery system researches are focused on cancer treatments, and there are more than a dozen journals that concentrate on this particular topic; however, commercially and widely used FDA-approved nanomedicines are limited to a few, including Abraxane for metastatic breast cancer, Doxil for ovarian cancer and Emend for chemotherapy induced nausea [1,2]. This dissonance in research effort and clinical usage of nanomedicine is often attributed to the biological consequences of using nanomaterials. In the past, the unique physiochemical properties of particles combined with the ability to modify materials at molecular length scales promised to solve many of the problems in drug delivery and improve clinical outcomes [3–10]. However, the issues involving the biological consequences of using nanomaterials caused the current complications in nanoscale drug delivery systems. Validating efficacy and establishing that there is no biotoxicity during drug design and development have been extremely challenging. This has been compounded by the fact that the research has been largely empirical and without the development of design rules, to slow down clinical applications of nanomedicine.

**Figure 1:**
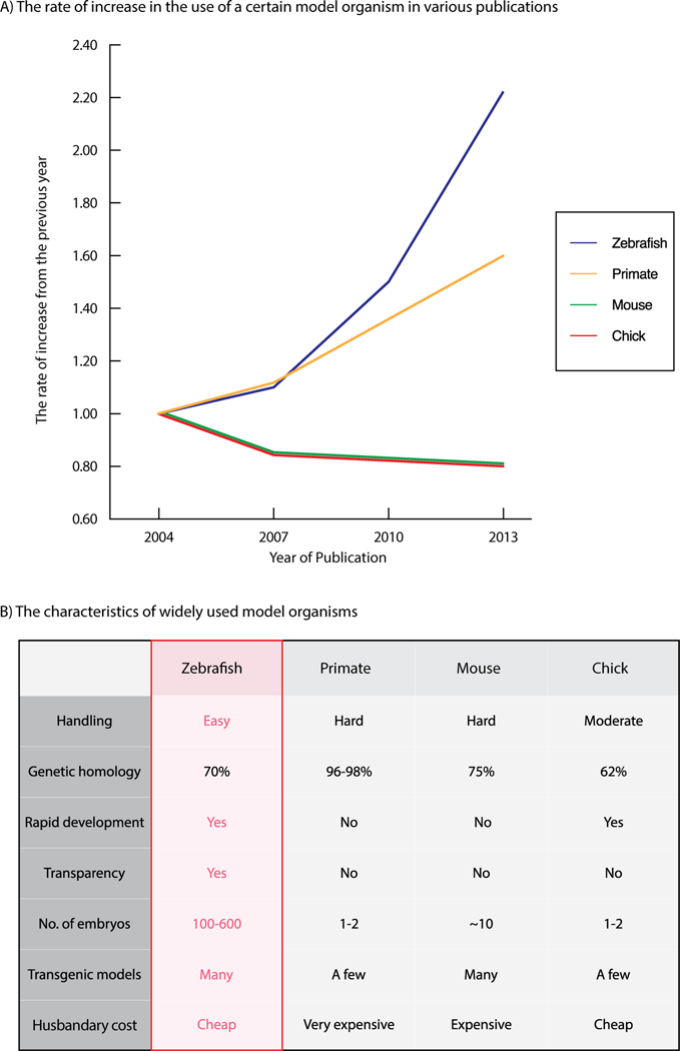
Comparison of the zebrafish with other in-vivo model organisms employed in biomedical-nano researches. (**A**) The prevalence of animal models in biomedical research. The normalized number of publications that employed zebrafish, primate, mouse, and chick models since 2004. The rapid growth of the zebrafish model highlights the benefits in preclinical studies. (**B**) The characteristics of widely used model organisms. Zebrafish, primate, mouse, and chick models are evaluated on important factors in preclinical studies. Apart from the genetic homology to humans, the zebrafish model has many advantages. The genetic homology of the animal model was obtained from Ensembl database browser analysis [11].

In designing and developing a new medicine for human, preclinical screenings with animal models are an essential part of gaining insight into the efficacy, biodistribution, and biotoxicity of nanomaterials [12–14]. Commonly, mouse models are used; however, preclinical screenings with animal models generally involve a large number of animals and hence can be expensive and time-consuming with mouse models (Figure 1B) [2,15–19]. Biological homologous to humans, primate models will provide the most reliable data [2,20–22]. However, experiments with baboons or chimpanzees are even more expensive and time-consuming (Figure 1B). Due to such high demand of money and time, many tend to refrain from including preclinical screenings with animal models during the development of new nanoscale drug delivery systems, and compromise with the less demanding *in vitro* assays.

The zebrafish model is an alternative animal model that can resolve the issue (Figure 1A) [2]. Zebrafish models are inexpensive, amenable to modifications, and optically transparent, and hence have many advantages over other animal models (Figure 1B) [2,6,13,16,23–43]. With zebrafish models, we can easily incorporate sophisticated preclinical screenings during the nanoscale delivery system development [13,23,24,30–32,34,38,44]. This review highlights the characteristics and uses of zebrafish models for the researchers who are not accustomed to experiment with them. Also, in order to guide them through toward adopting preclinical screenings with zebrafish models during their research and eventually to foster clinical application of nanoscale drug delivery system, we included a suggested guideline.

## Benefits and drawbacks of zebrafish models in preclinical studies of nanoscale drug delivery systems

### Benefits

#### Optical transparency

**Figure 2:**
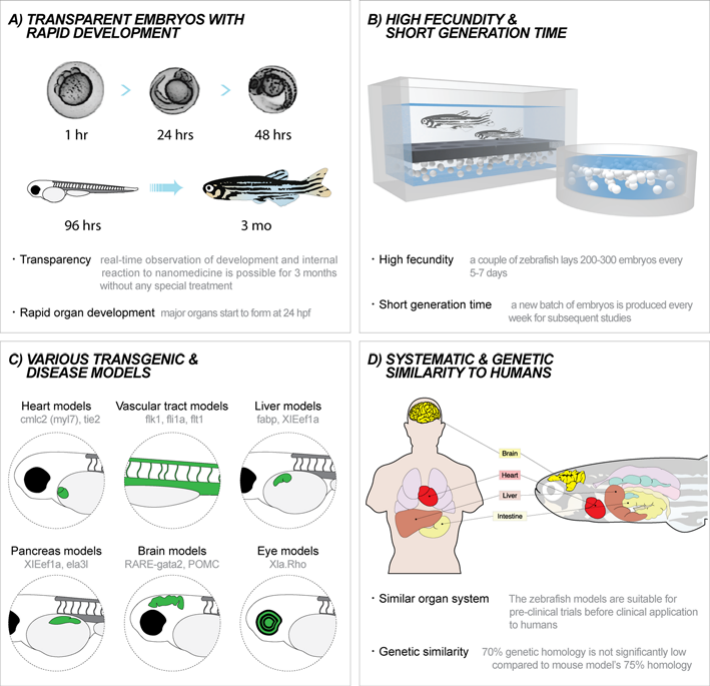
Characteristics of zebrafish models (**A**) Zebrafish are transparent and develop rapidly. Zebrafish embryos start forming major organs at 24 hpf. The transparency of zebrafish embryos is sustained until 96 hpf. Phenotypic changes upon exposure to drug delivery systems can be easily observed. (**B**) High fecundity and short generation time. A fertile zebrafish lays approximately 200–300 embryos every 5–7 days. Due to the high fecundity and short generation time, preclinical studies with zebrafish embryos can use large sample sizes, are cost-effective and can be conducted in a relative short time. (**C**) Numerous transgenic and disease models. Various models with fluorescent protein expressing organs are commercially available. Disease and knockdown models can be tailored for specific applications. (**D**) Systematic and genetic similarity to humans. Zebrafish models are phenotypically and genotypically similar to humans and hence suitable for prestudies. They have 70% genetic homology to humans, not significantly lower than widely used mouse models.

Zebrafish embryos retain optical transparency until approximately 60 h post fertilization (hpf), when the pigmentation process starts (Figure 2A) [24,45]. To keep the optical transparency beyond that point, transgenic models such as *casper*, which lacks pigments in its skin and scales, can be employed, or zebrafish embryos can be treated with 1-phenyl 2-thiourea (PTU) to inhibit pigmentation [24,46]. The transparency of zebrafish embryos offers unique opportunity to observe the events happening inside the body such as tumor development, vessel growth, organ development, and dispersion of nanomaterials. Such observations can be made after 96 hpf when major organs complete their development [24]. Due to relatively fast development as compared with other model organisms, preclinical screenings with zebrafish models can take less time because waiting period to notice significant changes in zebrafish’s behavior and morphology is shortened (Figure 2A) [31,32,45]. There are a wide range of transgenic zebrafish models with fluorescent organs that can help us to observe organ-specific reaction to nanomaterials through their clear body (Figure 2C) [34,44,46–49].

#### Statistical reliability

A fertile female zebrafish lays approximately 200–300 embryos every 5–7 days (Figure 2B) [30,31,50]. The high fecundity of zebrafish translates into statistical reliability, by providing large numbers of affordable subjects for preclinical studies. The development of new drug delivery systems requires extensive preclinical studies to support their efficacy and safety. This is particularly challenging with nanoscale delivery systems, which have been addressed with an issue in their safety. The productivity and relatively inexpensive price of zebrafish models enable us to design powerful preclinical studies with numerous experimental groups and large sample sizes, which can substantiate the capabilities and safety of a new nanoscale drug delivery system.

#### Feasibility for modification

In designing a powerful preclinical experiment, finding a suitable model can be extremely challenging. With zebrafish models, a large library of transgenic lines is commercially available. Most commonly adopted lines express fluorescent proteins on the surface of specific organs to facilitate the tracking of nanoscale drug delivery systems or examination of phenotypic changes upon exposure to them (Figure 2C). Moreover, zebrafish models can also be tailored at needs through genetic engineering, xenotransplanting, and injecting bacilli [34,45,46,51,52].

#### Regulation

The regulation of animal use in research is becoming more stringent [53,54]. For example, in Europe, animals cannot be tested after 7 days post-fertilization. Zebrafish embryos start forming major organs within 24 hpf, which allows researchers to observe biodistribution and biotoxicity early in development (Figure 2D) [24].

### Drawbacks

There have been factors that limited the use of zebrafish models. First, compared with other animal models such as mouse, it lacks an established protocol, which is a fact that significantly limits the ability to make meaningful comparisons and draw significant conclusions amongst different studies [55]. We hope that our suggested guideline can contribute to encourage the community to agree upon an established protocol for zebrafish models. Second, it is widely misunderstood that zebrafish’s low relevance to humans is not high enough for preclinical screenings of nanoscale drug delivery systems. However, in reality, zebrafish have similar organs and gene composition as humans. While mouse models have 75% genetic similarity to humans, zebrafish models have approximately 70% similarity [56].

## Functional screening of nanoscale drug delivery systems with zebrafish models

Nanomaterials’ versatility allowed the development of nanoscale drug delivery systems with ingenuous functionalities such as targetting ability and light/heat-reactive releasing capability of loads [57–60]. However, until those capabilities are proven to perfectly work in *in vivo* settings, the nanomedicine cannot be clinically adopted [61]. In functional screening of nanoscale drug delivery systems, researchers can choose from various transgenic and disease models of zebrafish to design a powerful tool to verify the efficacy and capabilities of their nanoscale drug delivery system.

### Transgenic models

#### Angiogenesis and anticancer drugs

As mentioned above, zebrafish models have transparent body to allow researchers to observe their blood vessels (BVs) and learn about the distribution and functionalities of nanoscale drug delivery systems [33]. Especially, because tumor development involves neovasculature, angiogenesis is an important indicator of tumor growth. Conversely, we can test the efficacy of our cancer treating nanoscale drug delivery systems by observing the inhibition of angiogenesis (Table 1) [62–66]. The BVs of zebrafish are readily observable by introducing fluorescent dyes [67,68]. Alkaline phosphate is the most widely used dye for staining the zebrafish embryos’ BVs [69,70]. This method has been used to observe the down-regulation of angiogenesis upon injection of an oncogenic signal pathway inhibitor (Table 1) [71]. If the dyeing process is too cumbersome, transgenic zebrafish lines with fluorescent BVs such as Tg(flk1:EGFP) [33,72] and Tg(flk1:mCherry) can be purchased (Table 1) [73].

**Table 1:** Preclinical studies of nanoscale drug delivery systems in the BVs of zebrafish models

Purpose	Drug delivery system	Vascular model	Cargo	Treatment method	Treatment time	Observation time	Ref.
**Inhibition of angiogenesis**	Polymeric micelle	Tg(flk1:mCherry)	Curcumin	Direct injection	48 hpf	10, 20, 40, 60 mpi	[73]
		Tg(flk1:EGFP)	Quecertin	Direct incubation	15 hpf; 54 hpf;	15 hpi; 48 hpi;	[74]
		WT	PI3K pathway inhibitor	Direct injection	48 hpf	48 hpi	[71]
		Tg(flk1:EGFP)	Curcumin	Direct incubation	48 hpf	120 hpf	[75]
**Tracking of nanomaterial**	Polymeric micelle	Tg(flk1:EGFP)	Doxorubucin	Direct injection	70 hpf	6 hpi	[76]
	Lipid nanodroplet	Tg(fli1:EGFP)	Cyanine dye	Direct injection	72 hpf	5–1 hpi	[77]

#### Organ models and organ-specific drugs

Transgenic models of zebrafish with fluorescent organs are readily available for tracking labeled nanoscale drug delivery systems or for testing their efficacy [34,44,78–81]. Tg(BMP4:EGFP) is a model with green fluorescent myocardium, that has been used for functional screening of common cardioactive and cardiotoxic drugs (epinephrine, doxazosin, esmolol, and doxorubicin). With more advanced imaging tools, pseudodynamic 3D images of the beating zebrafish heart were used to analyze changes in heart function following drug delivery [82]. Likewise, various organ models of zebrafish, for example those with fluorescent liver, brain, or pancreas, can be used to monitor organ-specific response to different nanomaterials (Figure 2C).

### Disease models

Although they have only been introduced to us a few decades ago, zebrafish models are starting to provide important insights into human disease because approximately 70% of zebrafish disease genes functionally match that of humans [66,83,84]. The most prevalent disease model is cancer model [69,71,74,85,86]*.* Moreover, various genetic engineering techniques or the uptake of bacilli can also induce various diseases in zebrafish models (Table 2) [87–92].

**Table 2:** Inducing disease in zebrafish models by injecting a xenograft or bacteria

Zebrafish model	Disease/cancer type	Method	Ref.
Tg(flk1:EGFP)	B16-F10 melanoma cells labeled with CM-DiI	Xenograft injection at 48 hpf	[74]
Tg(fli1:EGFP); Tg(mpeg1:mCherry); Tg(lyz;DsRed)	*Mycobacterium marium* (fish tuberculosis agent) with bacilli labeled with DsRed	Bacterial injection at 48–52 hpf	[87]
WT	MDA-MB-231/GFP; B16-F10 cells labeled with the QTracker Red kit	Xenograft injection at 48 hpf	[71]
WT	Methicillin-resistant strain of *Staphylococcus aureus*	Bacterial injection	[88]
rag2^E450fs^ mutant (casper)	GFP-labeled BRAF^V600E^	Xenograft injection at 3 months	[93]

#### Cancer models

There are several ways to produce cancer zebrafish models: chemical treatment, genetic technology, and xenotransplantation, the introduction of foreign tumor cells [66,84]. Exposure to dimethylbenzanthracene can induce intestinal cancer, and overexpression of *Myc* gene will cause T-cell leukemia in zebrafish [66]. Xenotransplantation can produce more various types of cancers compared with the other two methods [66,84]. The transplantation of human B16-F10 melanoma cells labeled with Qtracker red has been used to simulate human melanoma in zebrafish embryos (Table 2) [71]. Xenografts with fluorescent labels can be monitored after injection of nanoscale drug delivery system. Moreover, researchers could learn about metastasis by imaging in xenotransplanted zebrafish models by counting the number of circulating tumor cells, which is a good indicator for metastasis (Table 2) [74]. The growth of a transplanted GFP-labeled BRAF^V600E^ xenograft and subsequent vascularization around it were observed using flk1:mCherry; rag2^E450fs^ zebrafish models [93]. In addition, tumor heterogeneity can be investigated by distinguishing tumor cells by labeling with different fluorescent tags based on differentiation status. Similarly, crestin:EGFP zebrafish have been used to melanoma since crestin, which is expressed embryonically in neural crest progenitor cells, is re-expressed in melanoma [94]. They could observe the development of BRAF^V600E^ by tracking the expression of crestin over 40 days with p53/BRAF/crestin:EGFP zebrafish [94].

#### Other disease models

Our knowledge about zebrafish genome sequence allowed us to produce various disease models of it [83,92]. For example, metabolic diseases such as obesity, type 2 diabetes, and atherosclerosis can now be produced through genetic engineering, while in the past, we relied on less objective diet control methods. Moreover, because zebrafish share much of human brain’s circuitry [92], brain disorder studies with zebrafish models are also an emerging field [95]. Anxiety, depression, cognitive disorder, social disorder, ADHD, neurodegeneration, serotonin syndrome, eating disorders, and sleep disorders are common human brain disorders that can be studied through biobehavioral assays with zebrafish models, such as counting zebrafish subjects with lackluster locomotion [83,95].

Recently, transgenic zebrafish lines with fluorescent leukocytes, along with the discovery of reporter for the macrophage lineage, fostered the study of macrophage–pathogen interactions with zebrafish models. A team found their drug delivery system designed to treat tuberculosis was found to colocalize within the bacilli of macrophages by using Tg(mpeg1:mCherry) model with fluorescent macrophages [87]. The types of bacteria used for infecting zebrafish include *Salmonella typhimurium, Vibrio anguillarum, Bacillus subtilis, Listeria monocytogenes, Mycobacterium marinum, Burkholderia cenocepacia, S. aureus, Shigella flexneri, and Candida albicans* (Table 2) [91,96].

## Toxicity screening for nanoscale drug delivery systems with zebrafish models

**Figure 3:**
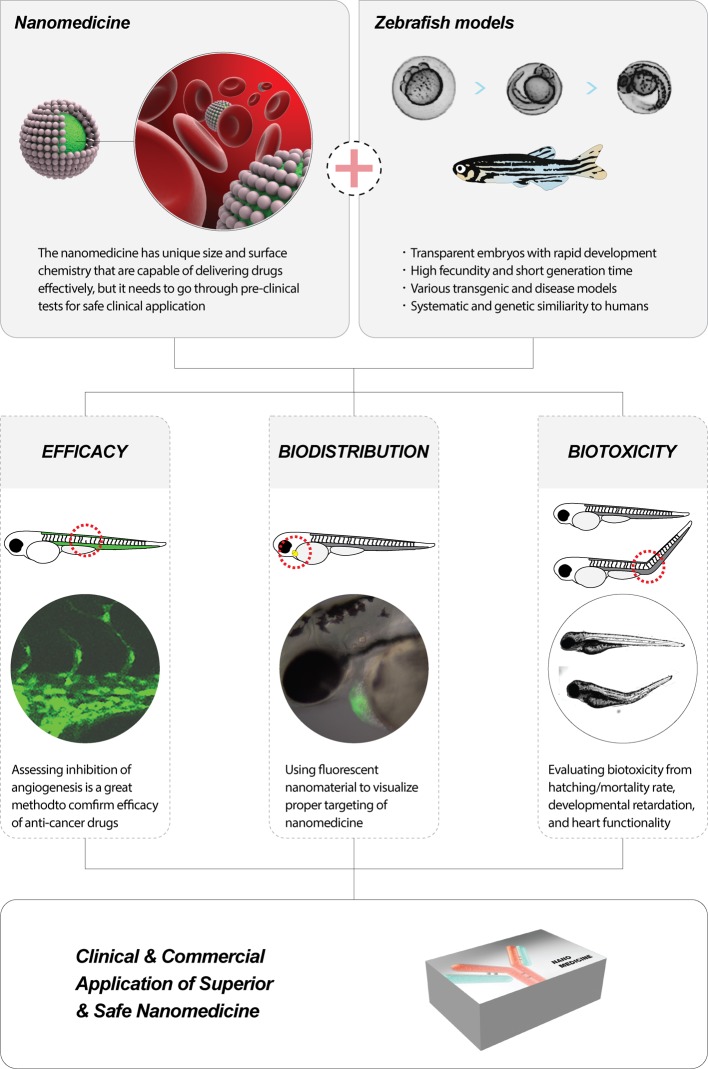
Zebrafish models for preclinical studies of nanoscale drug delivery systems (**A**) Therapeutic biology researches to study efficacy of anticancer treatments using zebrafish. (**B**) Biodistribution researches to study through tracking and imaging with fluorescent nanomaterials using transparent zebrafish embryos. (**C**) Biotoxicity researches within the zebrafish to evaluate and resolve concerns regarding toxicity of nanomaterials.

Any new drug delivery system is of no use if it is not safe. Therefore, many successfully funded nanoscale drug delivery system development processes often include exhaustive toxicity screening with mouse models. However, preclinical studies with mouse models can be extremely time and labor consuming, and moreover, expensive for most research labs. *In vitro* toxicity assessments, which they often compromise for testing the safety of their nanoscale drug delivery systems, cannot mimic the exposure in human body (Table 5). By adopting toxicity screening with less demanding zebrafish models, we can foster the community to produce more biosafe nanoscale drug delivery systems (Figure 3) [2].

Zebrafish is an ideal model for the study of toxicity screening because a zebrafish embryo mimics the complexity of interactions in the human body. With a high degree of homology to human genome and biological systems, zebrafish embryos can be used to monitor the phenotypic and genotypic abnormalities upon exposure to nanomaterials. Observing change in the size and shape of organs during development is simple yet valuable indicators of biotoxicity. Low husbandry cost, rapid development, and ease in handling allow rapid high throughput and cost-effective preclinical screenings [2,97].

Unfortunately, even among the nanoscale delivery system studies that used zebrafish as animal models, only a few of them included the toxicity screening with zebrafish models. (Table 5). Most commonly, the embryos’ hatching rates and survival rates were observed at 72 hpf. Phenotypic deformations were monitored after 4 h post-incubation with different concentrations of nanomaterials in 24-wells. The aberrations in tail length, poor pigmentation, tail bending, and organ defects at higher concentrations represented phenotypic deformities due to drug delivery systems [97–100]. Nadrah et al. [99] applied additional assessments during 24 hpf through 47 hpf, which included observing characteristics such as egg coagulation, missing heartbeat, missing somites, missing tail detachment from the yolk sac, no eye and body pigmentation, missing blood flow, spine deformation, yolk sac edema, and incomplete eye and ear development. Uniquely, Daniel et al. [101] fed chironoma incubated with silver nanoparticles to study toxic effects and biodistribution of silver nanoparticles. Also by using whole body X-ray and UV-Visible fluorescence spectrum, they demonstrated that the silver nanoparticles penetrated the blood–brain barrier to reach the brain.

Investigating intracellular fate of nanomaterials in zebrafish can be done via TEM analysis of ultrathin sections of zebrafish subjects (Table 3) [26,66,102]. He et al. used the micrographs ultrathin sections of muscle cell to observe the intracellular deposits of S-TiO2 nanoparticles [26] The micrographs showed both lysosome and endosome formation, which indicated that the nanoparticles were introduced through receptor-mediated mechanism rather than simple diffusion [26]. Muth-Kohne et al. [66] also confirmed the cellular uptake of Ag nanoparticles by the lumen of developing gut and erythrocytes through TEM analysis. Sectioning specific organs such as kidney can offer insights into bioaccumulation and toxicity at specific organs as well. TEM analysis can be used to confirm the uptake of drug delivery systems and their uptake mechanisms.

**Table 3:** TEM analysis of zebrafish models

Nano-particle	Method	Concentration	Treatment time	Observation time	Ref.
S-TiO2	Suspension	100 ppm	4–6 hpf	120 hpf	[26]
AgNP	Suspension	4 and 16 mg/l	1 hpf	48 hpf	[66]
Ag-loaded alumina, Au-loaded alumina, Pt-loaded alumina	Suspension	100, 200, 500, 1000 μg/ml	6 hpf	96 hpf	[102]

Inductively coupled plasma MS (ICP-MS) is another way to confirm and quantitate the uptake of nanoscale drug delivery system by zebrafish embryos (Table 4) [83,92,95,96]. Elemental contents of the zebrafish embryos are analyzed by fixing and dissolving the embryos in a solution. For example, Zarco-Fernandez et al. [83] monitored Cd concentration in the embryos to quantitate quantum dot uptake.

**Table 4:** ICP-MS analysis of zebrafish models

Nanoparticle	Method	Treatment time	Observation time	Element	Ref.
QD	Suspension	72 hpf	120 hpf	Cd	[83]
AgNP	Suspension	6 hpf	72 hpf	Ag	[96]

**Table 5:** Models and methods used for toxicity screening in nanoscale drug delivery system research with zebrafish models

Model	Method	Drug delivery system/nanomaterial	Drug/cargo	Ref.
**Zebrafish embryos**	Direct incubation	Ag nanoparticles	N/A	[101]
		Au nanoparticles	N/A	[98]
		Mesoporous silica nanoparticles capped with β-cyclodextrin	N/A	[99]
		Hydroxyapatite nanoparticles	N/A	[100]
		Au nanoparticles	6-mercaptopurine	[97]
		Graphene nanoparticles	Curcumin	[103]
	Direct injection	Ag nanoshell	N/A	[104]
		Graphene nanoparticles	N/A	[105]
		Lipid nanodroplet	Cyanine dye	[77]
		Polymer nanoparticles	LY294002	[72]
**Cells**	MTT assay	Polymicelles	Curcumin	[75]
		Polymicelles	Doxorubicin	[76]
		ZnO micronanostructures	N/A	[106]
		Lipid nanodroplet	Cyanine dye	[77]
		Graphene nanoparticles	Curcumin	[103]
		Polymer nanoparticles	LY294002	[71]
		PEG PCL nanoparticles	Quecertin	[74]
**N/A**		Graphene nanoparticles	GFP	[107]
		Silica-based colloidosomes	All *trans-*retinoic acid	[108]
		Iron oxide nanoparticles	Cisplatin	[109]
		Copolymer micelles	Curcumin	[73]

Only some studies that used zebrafish models for studying efficacy and biodistribution kept the same model for toxicity screenings. A few studies used *in vitro* model, and few omitted the toxicity screening. **Bold**: Subjectts of toxicity screening

## A guideline for preclinical screenings of nanoscale drug delivery system with zebrafish models

We compiled a list of toxicity screening methods employed by papers that used zebrafish models for testing capabilities of their respective nanomaterials (Table 6). It was frustrating to notice that more than half of the papers did not perform the toxicity assessments on zebrafish models despite their knowledge about it. In order to foster future papers to include simple yet powerful assays to confirm the safety of their nanoscale drug delivery systems on a living organism, we benchmarked different papers to suggest various toxicity screening assays using zebrafish embryos (Table 6).

**Table 6:** Proposed recommendations for preclinical studies of nanoscale drug delivery systems with zebrafish models

	Direct incubation	Direct injection
**Treatment time point**	6 hpf	48 hpf
**Observation time point**	48, 72, 96, 168 hpf	1, 24, 48, 72, 96, 120 hpi
**Hatching/mortality rate observation time point**	72 hpf	
**Heartbeat observation time point**	48, 72, 96 hpf	
**Biodistribution observation**	TEM analysis	
**Nanomaterials uptake quantitation**	ICP-MS analysis	

**Bold**: the time points for certain actions in preclinical studies Abbreviation: hpi, h post injection.

In direct incubation method, the zebrafish embryos were exposed to the solutions of nanomaterials at 6 hpf [68,110–112]. Upon incubation, the biodistribution and efficacy of nanoscale drug delivery systems can be monitored at 48, 72, 96, and 168 hpf. For direct injection method, nanomaterials can be injected to the embryos at 48 hpf, after completing vascular development [68,113]. First observation can be made at 1 h post injection, and subsequent observations can be made at 24-h intervals for long-term effects.

The mortality and hatching rates of zebrafish embryos are the most commonly and conveniently monitored indicators of the biotoxicity. Both factors are measured at 72 hpf when the embryos normally hatch [110–112,114]. Milder toxic effects are usually reflected through heart defects, which can be assessed by monitoring the heart rate [24,45,110–112]. We recommend counting heartbeats for a 30-s period at 48, 72, and 96 hpf when the heart is fully developed. Additionally, liver, the site of nanomaterial accumulation, can be monitored from 96 hpf with 24-h intervals.

## Conclusion

Functional and toxicological screenings of nanomaterials are essential for successful development of nanoscale drug delivery system, especially when the safety of nanomedicine is continuously questioned. However, many related studies fail to include preclinical studies with animal models because they normally cost considerable labor, money, and time. Zebrafish is a great alternative animal model, wrongly perceived to have poor homology to humans. However as mentioned above, zebrafish share most of disease genes and biological systems with humans. Moreover, the advantages of zebrafish model make it a versatile animal model for nanomedicine research.

Last, recent studies with zebrafish models are performed under a wide range of experimental conditions making comparison and benchmarking almost impossible. Therefore, we included a guideline for people who want to adopt zebrafish models into their research. We hope that in the future, a protocol for zebrafish will be established to accelerate the clinical use of nanoscale drug delivery systems.

## Abbreviations

BV: blood vessel
hpf: h post fertilization
ICP-MS: inductively coupled plasma MS
